# Response: Commentary: Toward a more physiologically and evolutionarily relevant definition of metal hyperaccumulation in plants

**DOI:** 10.3389/fpls.2015.01252

**Published:** 2016-01-08

**Authors:** Eric W. Goolsby, Chase M. Mason

**Affiliations:** ^1^Department of Plant Biology, University of GeorgiaAthens, GA, USA; ^2^Interdisciplinary Toxicology Program, University of GeorgiaAthens, GA, USA; ^3^Arnold Arboretum, Harvard UniversityBoston, MA, USA

**Keywords:** hyperaccumulation, tolerance, physiology, manipulative experiments, metalliferous soils, adaptationism, function-valued traits

## Introduction

In our opinion piece (Goolsby and Mason, 2015), we highlighted several problems with the current ecological definition of plant metal hyperaccumulation as applied to physiological, genetic, and evolutionary perspectives, and put forward a more compatible definition based primarily on how plant responses to other abiotic stresses are defined. The commentary by Van Der Ent et al. (2015) mischaracterizes several important issues and continues to advocate for a “phenomenological” definition of hyperaccumulation which confounds metal uptake, tolerance, and geography.

From an intuitive standpoint the word “hyperaccumulation” means the collection of large quantities of a substance, here metals. The capacity to take up and concentrate metals in shoot tissue is first and foremost a physiological trait. All abiotic stress responses are complex, but the ability of a plant to respond to the abiotic environment is an inherent property of that plant's genetic makeup and resulting physiology, and plant traits are not normally defined by the geography of where a plant grows in nature. Manipulative experiments that identify important plant responses to salt, drought, temperature, or low-nutrient stress are not dismissed because the plant under study does not typically occur in environments that experience the stress in question.

Heavy metal hyperaccumulation is not justifiably different, and there is no reason for researchers working on the evolution of this fascinating trait to abide by an overly restrictive definition that does not well-describe the physiological trait under study. The definition of hyperaccumulation emphasized by Van Der Ent et al. (2013, 2015) starts with the reasonable consideration of the level of uptake and accumulation of metals in shoot tissue, then confounds this intuitive definition with the requirement of a separate physiological trait (tolerance of high soil metal concentration), and further adds the ecological and geographical requirement that plants form natural populations on metalliferous soils. This definition by its very nature discourages a wide variety of high-quality manipulative research approaches into the evolution of hyperaccumulation capacity, as any plants not found in metal-rich environments are excluded from being “hyperaccumulators” regardless of their inherent hyperaccumulation ability, and attempts to dissect the evolution of the separate traits of tolerance and hyperaccumulation are muddled. Fortunately, researchers are already independently rejecting the assumptions of this definition (e.g., Cappa et al., 2015).

Space restrictions on this article type prevent us from thoroughly addressing all of the inaccuracies in the Van Der Ent et al. (2015) commentary, so we specifically focus on the most egregious misrepresentations that are most relevant to the study of the evolution of hyperaccumulation below. We encourage readers to investigate the validity of other claims made in the commentary, including those related to the discovery of new hyperaccumulator species not known from natural metalliferous soils in anthropogenically contaminated habitats (e.g., *Silene latifolia*, Escarre et al., 2011), the ability of crop species to accumulate metals in excess of thresholds without toxicity under relatively low soil metal concentrations (meeting our definition of hyperaccumulation that removes the separate trait of tolerance—e.g., sunflower, (Cutright et al., 2010); amaranth, (Fan and Zhou, 2009); and bok choy, Sudmoon et al., 2015), and whether natural soils are in any way superior to amended soils with respect to understanding and controlling soil metal bioavailability and equilibrium, given that using standardized sufficiently equilibrated amended soils in a greenhouse setting provides a fair comparison across relative levels of metal availability.

“*…the key characteristic of hyperaccumulator plants is their highly efficient uptake behavior and a non-linear response to soil trace element concentrations*…  *There is no evidence that pot-grown hyperaccumulator plants quickly exhaust the available metals in the soil in most experimental settings, as alleged by Goolsby and Mason*.”

**Figure 1:**
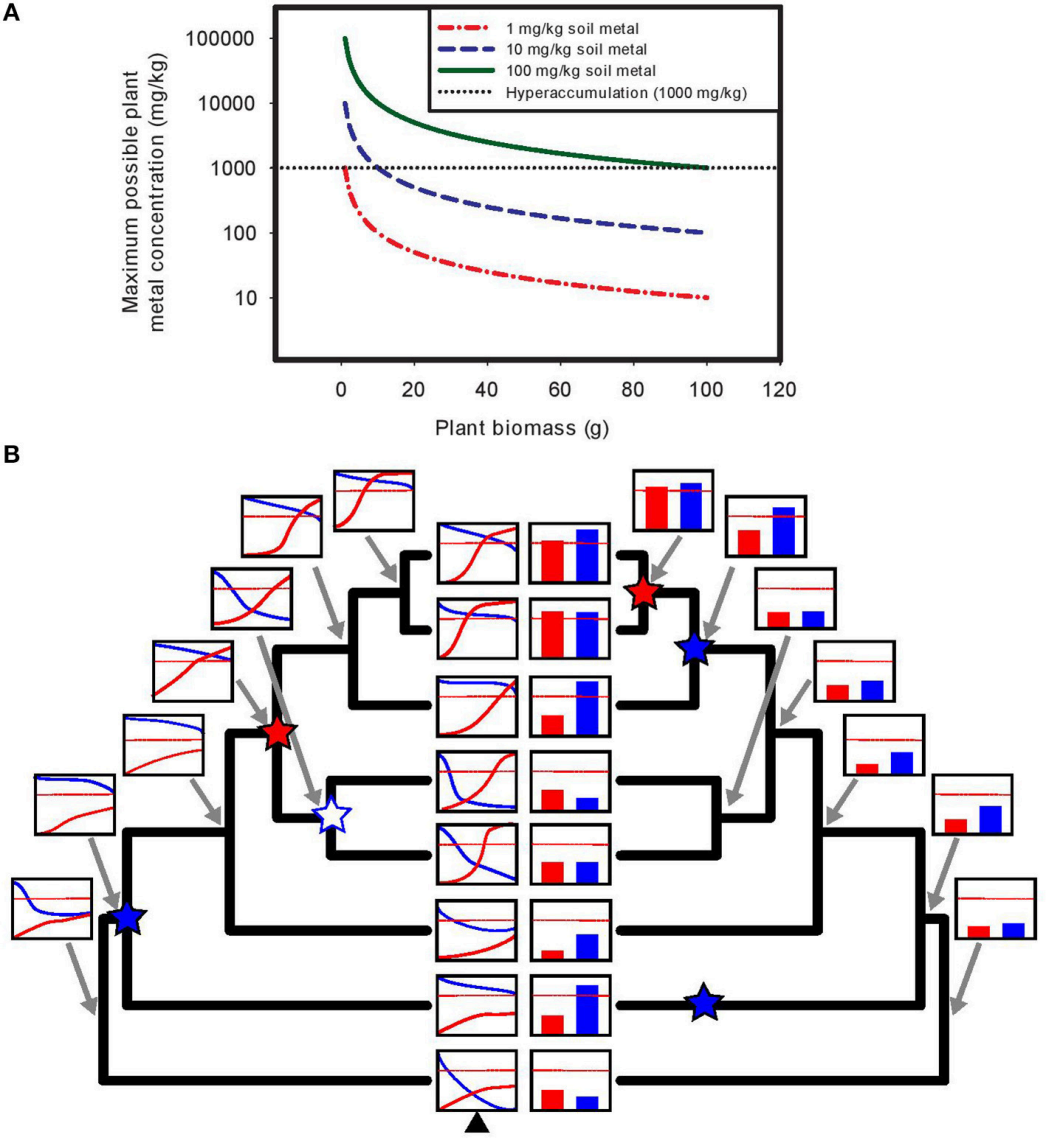
**Demonstration of the mathematical impossibility of achieving hyperaccumulation in pots using soils with low metal concentrations, and the utility of understanding plant responses across experimental soil metal gradients. (A)** In experiments assessing hyperaccumulation ability in pots, using soils with low metal concentrations results in rapid exhaustion of available soil metals as plants grow and increase in mass. In the example shown, a plant is grown in a pot filled with 1 kg of soil containing a soil metal concentration of either 1, 10, or 100 mg/kg, and the maximum possible plant metal concentration (if the plant were able to take up every metal ion in the soil) is plotted as a function of total plant biomass. Even with complete metal uptake, achieving hyperaccumulation thresholds is mathematically impossible for plants that grow to any appreciable size (e.g., reproductive stage in all but the very smallest species, like perhaps *Arabidopsis*). At 1 mg/kg of soil metals, reaching the hyperaccumulator threshold is essentially impossible regardless of plant size, while at 10 mg/kg it is impossible for plants growing to more than 1% of the soil mass in the pot, due to rapid dilution of accumulated metals across total plant biomass. The use of soil metal amendments (or “spiked” soils, e.g., 100 mg/kg in 1 kg of soil) is often necessary to detect hyperaccumulation ability in pot experiments. **(B)** Using experimental soil metal gradients provides a powerful approach to untangling the evolutionary dynamics of the separate traits of hyperaccumulation and tolerance. Assessing these traits at a single soil metal concentration can result in highly biased ancestral reconstructions due to the nonlinearity and threshold effects inherent in plant responses to soil metals. In the example shown (left tree), dose-response curves of metal accumulation (e.g., shoot metal concentration, in red) and metal tolerance (e.g., biomass or fitness, in blue) are generated using assessment over an experimental gradient of soil metal concentrations (x-axis), and ancestral reconstruction of these curves is performed (e.g., via the methods of Goolsby, 2015). The red dotted line represents hyperaccumulation threshold criteria. Using ancestral curve reconstruction (left tree), a single origin of hyperaccumulation (red star) is correctly identified, along with a single origin of tolerance (blue star) and one subsequent loss of tolerance (white star with blue outline). By contrast, assessing plant responses at a single soil metal concentration (here the soil metal concentration represented by the black triangle at the bottom of the left tree) captures only a snapshot of plant responses to metals (bar graphs, right tree). Using ancestral reconstruction based on such a narrow snapshot (right tree), one incorrectly infers a very different evolutionary history of hyperaccumulation and tolerance.

Assuming a non-linear response to soil metal concentrations, examining uptake and tolerance at a single soil metal concentration (e.g., natural soil collected from a site) is unreliable, whereas examining uptake patterns along a controlled soil metal gradient can definitively identify both accumulation and tolerance (see Figure 1B). Furthermore, it is often mathematically impossible to achieve metal hyperaccumulation using soils with low metal concentrations in pots, particularly if the requirement that hyperaccumulation be demonstrated at seed set is imposed. As plants grow and shoot mass increases, the finite amount of metals available is easily diluted below hyperaccumulator thresholds, even if plants could be perfectly efficient and remove all metal ions from the soil volume (see Figure 1A).

“*Is there really a near-impossibility of disentangling tolerance and accumulation if only naturally-occurring hyperaccumulator plants are used?*”

Yes! In order to disentangle the evolution of tolerance and accumulation across a group of species, the most definitive approach is the manipulative construction of dose-response curves of accumulation and tolerance across gradients of metal concentration in each species, followed by the use of phylogenetic comparative methods for the reconstruction of the evolution of these traits (Goolsby, 2015). Such approaches are incredibly powerful and are key to understanding the order in which these two traits evolve, as well as the rigorous assessment of multiple evolutionary hypotheses (e.g., elemental defense, drought resistance, etc.).

“*If there is no ecological function or advantage, there is no demonstrable evolutionary relevance, because natural selection can only act on traits that are expressed in nature, not on ‘artificially-inducible phenotypes.’ Extreme natural phenotypes such as hyperaccumulators must have evolved through positive, directional natural selection, rather than neutral genetic drift*.”

It is well-accepted in evolutionary biology that complex phenotypes often arise as a result of genetic drift, indirect selection on other traits, and combinations thereof. While many species of metalliferous habitats have likely evolved hyperaccumulation in response to metal stress, many species known to exhibit the capacity for hyperaccumulation are not known to be native to natural metalliferous habitats. For instance, the authors of the commentary admit that the hyperaccumulation of manganese in *Phytolacca americana* is thought to have evolved as a side effect of the evolution of phosphorus-acquisition strategies in non-metalliferous soils (Lambers et al., 2015). Indeed, multiple hypotheses explaining the evolution of hyperaccumulation invoke side effects of mechanisms conferring resistance to drought, salt, or low-nutrient conditions. The assertion that hyperaccumulation has solely (or even primarily) evolved in response to selection from metalliferous habitats is highly disputed in the literature. In fact, phylogenetic comparative analyses of the distribution of hyperaccumulation suggest this may be a broadly ancestral latent trait in angiosperms (Cappa and Pilon-Smits, 2014). The relative rarity of metalliferous habitats in relation to the abundance of arid, infertile, and saline habitats suggests that indirect selection may play a larger role in hyperaccumulation evolution than is currently appreciated.

Problematically, these perspectives are apparently shared by three of the four authors of the commentary (Van Der Ent et al., 2015), who together published parallel conclusions just last year—“*Several hypotheses suggested to explain the evolution of hyperaccumulation seem unlikely when most populations of a species occur on normal soil, where plants cannot hyperaccumulate due to low metal availability. In such species, it may be that hyperaccumulation is an ancestral phylogenetic trait or an anomalous manifestation of physiological mechanisms evolved on normal soils, and may or may not have direct adaptive benefits*” (Pollard et al., 2014). This paper actually provides multiple examples where directional selection on metalliferous soils is unlikely to have driven the evolution of hyperaccumulation, and provides multiple alternative explanations. If “facultative hyperaccumulators” found both on and off metalliferous soils are predicted to have evolved their hyperaccumulation ability on normal soils, why should there not be myriad other undiscovered species with hyperaccumulation ability that have never colonized metal-rich habitats?

“*In some cases it may be appropriate to study metal uptake in species from non- metalliferous soils, but only when driven by specific hypotheses such as comparisons among conspecific metallophytes and non- metallophytes in a primarily hyperaccumulating lineage*”

The rationale for restricting investigation of a physiological trait based on plant geography and source ecology is inexplicable. As we have explained, hyperaccumulation has been strongly suggested to evolve as a by-product of selection for physiological processes unrelated to adaptation on metalliferous soils. The rationale that restricts the study of metal hyperaccumulation to species from metalliferous soils would have plant biologists only study salt tolerance in species from haline environments, drought tolerance in species from arid environments, and nutrient stress only in species from infertile environments. Obviously, this is an untenable restriction that is rejected by researchers who study other forms of plant stress. Furthermore, given the suggestion from phylogenetic analyses that hyperaccumulation may be a widespread latent trait in plants (Cappa and Pilon-Smits, 2014; Pollard et al., 2014), such restrictions will severely limit our ability to understand the evolution of heavy metal hyperaccumulation.

### Conflict of interest statement

The authors declare that the research was conducted in the absence of any commercial or financial relationships that could be construed as a potential conflict of interest.
